# Anal signs of child sexual abuse: a case–control study

**DOI:** 10.1186/1471-2431-14-128

**Published:** 2014-05-27

**Authors:** Christopher J Hobbs, Charlotte M Wright

**Affiliations:** 1 St James’s University Hospital, Leeds, UK; 2 PEACH Unit, School of Medicine, MVLS College, University of Glasgow, Glasgow, UK; 3 Yorkshire Medicolegal Chambers, Albion Mills, Albion Road, Greengates, Bradford BD10 9TQ, UK

**Keywords:** Child abuse, Sexual, Forensic medicine, Community child health

## Abstract

**Background:**

There is uncertainty about the nature and specificity of physical signs following anal child sexual abuse. The study investigates the extent to which physical findings discriminate between children with and without a history of anal abuse.

**Methods:**

Retrospective case note review in a paediatric forensic unit.

Cases: all eligible cases from1990 to 2007 alleging anal abuse.

Controls: all children examined anally from 1998 to 2007 with possible physical abuse or neglect with no identified concern regarding sexual abuse. Fisher’s exact test (two-tailed) was performed to ascertain the significance of differences for individual signs between cases and controls. To explore the potential role of confounding, logistic regression was used to produce odds ratios adjusted for age and gender.

**Results:**

A total of 184 cases (105 boys, 79 girls), average age 98.5 months (range 26 to 179) were compared with 179 controls (94 boys, 85 girls) average age 83.7 months (range 35–193). Of the cases 136 (74%) had one or more signs described in anal abuse, compared to 29 (16%) controls. 79 (43%) cases and 2 (1.1%) controls had >1 sign. Reflex anal dilatation (RAD) and venous congestion were seen in 22% and 36% of cases but <1% of controls (likelihood ratios (LR) 40, 60 respectively), anal fissure in 14% cases and 1.1% controls (LR 13), anal laxity in 27% cases and 3% controls (LR 10).

Novel signs seen significantly more commonly in cases were anal fold changes, swelling and twitching. Erythema, swelling and fold changes were seen most commonly within 7 days of last reported contact; RAD, laxity, venous congestion, fissure and twitching were observed up to 6 months after the alleged assault.

**Conclusions:**

Anal findings are more common in children alleging anal abuse than in those presenting with physical abuse or neglect with no concern about sexual abuse. Multiple signs are rare in controls and support disclosed anal abuse.

## Background

Child Sexual Abuse (CSA) diagnosis has been likened to a “jigsaw puzzle” [1]. Whilst the child’s allegation is vital, physical evidence obtained by an appropriately qualified examiner [2] can support criminal prosecution and child protection. Physical evidence has been the subject of consensus statements [3] and systematic review [4]. Anal findings are described following CSA [5-13], in children selected for non-abuse [14-16] and those with medical conditions affecting the anus [17-21]. There have been two previous studies where anal signs in different groups of children were compared [10,11].

If present, anal signs may be used in children with a disclosure of CSA to provide corroboration for court proceedings, but it is not currently clear how much reliance can be placed on which signs. There is even less certainty about the extent to which anal signs seen in children with no disclosure or suspicion should raise concern about possible CSA and the need for further investigation.

This study aimed to compare the prevalence of anal findings as assessed by specialist forensically trained paediatricians in a group of children where the history included a statement by the child of anal abuse with a group of children with a history solely of non-sexual physical abuse or neglect and with no concerns re sexual abuse.

## Methods

Cases and controls less than 16 years of age were identified retrospectively from a paediatric forensic centre in Leeds, a metropolitan city in Northern England. Children referred by social services or police are usually seen within 72 hours, either before or after formal interview [22]. All medical reports are held on a dedicated electronic database including digital clinical images since 2001. It was routine practice at this time to photo document examination findings in all children examined for forensic purposes.

### Case selection

#### Cases

These were all children on the data base who had made a specific disclosure of anal abuse investigated by statutory agencies. The reports database was searched using the phrases “anal abuse” and “anal penetration” between 1990 and 2007. In addition all children with genital/anal photographs in the clinical images database were identified. This enabled an additional smaller group of cases missed by the key word search to be identified, the reports of these children having been checked manually.

#### Controls

These were all children referred to the same centre for suspected physical abuse or neglect of themselves or siblings who had forensic inspection of the anus in accordance with national guidance [23], and in whom CSA was excluded as far as possible. There were more controls than cases so only children seen between 1998 and 2007 and aged 3 years or over were used. Potential controls were identified in the database by using the phrase “non-accidental injury” then manually searching the reports for evidence of an anal examination. In addition a further larger group of control children with anal photographs were identified via the clinical images database over the same time period and this included non-index siblings and neglected children.

Children were excluded as controls if there had been any suggestion in the report of:

1. Allegation of sexual abuse

2. Sexualized behavior

3. Current or past contact with known or suspected sexual offender

4. Current or past concern from referring agency re CSA in child or sibling

5. Sexually transmitted infection

6. Presentation with gross genital or anal injury (from either an alleged accident or abuse)

7. Medical condition potentially affecting the anus e.g. Crohn’s disease, severe chronic constipation, myotonic dystrophy

Children with mild constipation or soiling were included as controls as these symptoms were not uncommon in children whether abused or non-abused. No exclusion was made on the presence of physical signs (anal or otherwise) whether or not suspected to be related to CSA.

#### Examination procedure

Examinations were undertaken by paediatricians specially trained in assessment of suspected CSA working in a team. Specialist paediatric registrars in training were supervised by an experienced forensically trained consultant paediatrician.

Anal inspection was routinely undertaken in the left lateral position without digital or instrumental examination. Buttock separation was maintained for 30 seconds to allow anal dilatation to occur when present. A standard examination proforma encouraged detailed recording of history and examination. Olympus and Zeiss colposcopes with 35 mm cameras (film and digital) were used.

Physical signs were confirmed either at joint medical examination or by review of photographic records or both. Cases were discussed at weekly departmental meetings and reports and photographs peer reviewed monthly.

#### Data retrieval

Details of the allegation, anal findings and constipation history were extracted from medical reports and entered, anonymized, onto an access database. Signs were described according to definitions in Table 1. Estimated diameter of reflex anal dilatation (RAD) was recorded when present.

**Table 1:** **Definition of anal physical signs used in this study**[4,9,24]

**Signs summarized in RCPCH systematic review**	
Reflex Anal Dilatation	The dynamic observation of the anus opening after minimal buttock traction, with relaxation of the external and internal sphincter muscles.
Laxity	Decreased anal muscle tone. This is a static findings; the diameter does not change upon inspection.
Gaping	An anus which, on separation of the buttocks, is already dilated, with a view into the anal canal or rectum, and remains so for the duration of the examination. This is a static sign. Anal gaping is of greater degree than anal laxity
Fissure/laceration	A break (split) in the perianal skin which radiates out from the anal orifice which may be superficial or deep
Reddening	Redness of the skin and/or mucous membranes caused by dilatation of the underlying capillaries
Perianal venous congestion	The collection of venous blood in the venous plexus of the perianal tissues creating a flat or swollen purple discoloration that may be localized or diffuse. It is distinct from bruising
Tag	A protrusion of anal verge or perianal skin, which interrupts the symmetry of the perianal skin folds.
Scar	Fibrous tissue that replaces normal tissue after the healing of a wound.
Bruise	A localized collection of blood in the skin and or subcutaneous tissue occurring as a result of damage to the capillaries or larger blood vessels allowing blood to leak into the tissues leading to skin discoloration
**Novel/other signs**	
Twitching anus	Rapid contraction and relaxation of the anal sphincter without dilatation
Swelling (“tyre sign”)	Swelling of the perianal area, giving appearance of a tyre
Funnelling	A deep-set or dished anal appearance, but without full dilatation
Abrasion	A superficial injury involving only the outer layers of the skin/mucous membrane that does not extend to the full thickness of the epidermis.
Mucosal Prolapse	Rectal mucosa extending down through a dilated anal sphincter
Anal Verge Deficit	A defect or gap in the tissue overlying the subcutaneous external anal sphincter at the most distal portion of the anal canal (anoderm) which extends exteriorly to the perianal skin.
Fold Change	Unusual, irregular or asymmetrical folding of the perianal skin radiating from the anal verge
Soiled	The presence of significant quantities of faeces around the anus

Ethical approval was obtained from Leeds (East) Health Service Ethics Committee (reference number 08/H1306/106).

### Analysis

Statistical analysis was performed using SPSS 14.0 for windows. Fisher’s exact test (two-tailed) was performed to ascertain the significance of differences for individual signs between cases and controls. Likelihood and odds ratios for cases versus controls were calculated for all signs. The likelihood ratio for any sign is the ratio of the percentage of cases showing the signs to the percentage found in the controls [25]. For signs not found at all in controls, to avoid division by zero, one dummy female control with mean values for age and date was added who was positive for all those signs. To explore the potential role of confounding, logistic regression was used to produce odds ratios adjusted for age and gender.

## Results

A total of 19,785 children were seen and reported for child protection concerns in Leeds from January 1990 to December 2007, of whom 3,119 were categorized by the examining doctor as likely CSA. From these, 184 cases (105 boys, 79 girls) were identified with disclosure by the child of anal abuse, mean age 98.5 months, range 26 to 179 months, with only 7 younger than 3 years; 142 were identified from main database, 42 via the photographic database. There were 179 controls (94 boys, 85 girls, average age 83.7 months (range 35–193) from 1998 to 2007; 76 identified from the main database, 103 from photographic database.

Thirteen permanent paediatric staff examined 136 cases (74%) and 100 controls (56%) of whom three examined 35% of cases and 31% of controls. The remainder were examined by trainees supervised by forensically trained paediatricians.

In 134 cases where an object was specified, alleged penetration was penile for 64% (86) and digital for 30% (41). A majority of cases (74%) had one or more core Royal College of Paediatrics and Child Health signs [4] (Table 2) and 43% two or more, compared to only 16% and 1% of controls respectively.

**Table 2:** Frequency of classic signs associated with anal abuse in cases and controls

						**Unadjusted**		**Adjusted+**	
**Sign**	**Cases**		**Controls**		LR*	**OR**	**P**	**OR**	**95% CI**
Reflex anal dilatation	41	22%	0		40.1	51.3	<.0001	62.35	8.4 - 462
Gaping	5	2.7%	0		4.9	5.0	0.12		
Laxity/reduced anal tone	49	27%	5	2.8%	9.6	4.9	<.0001	13.7	5.3 - 35.8
Reddening/Erythema	56	30%	15	8.3%	3.6	4.9	<.0001	5.3	2.8 - 10.0
Perianal venous congestion	66	36%	1	0.6%	59.8	99.6	<.0001	101	13.8 - 743
Fissure/laceration	26	14%	2	1.1%	12.8	14.6	<.0001	13.5	3.1 - 58
Tag	8	4.3%	10	5.6%	0.8	0.8	>0.5		
Scar	10	5.4%	0		9.0	10.3	0.002	8.2	1.0 - 66.4
Anal or perianal bruising	0		0						
None of the above signs	48	26%	150	84%	0.31	0.07	<.0001	0.059	0.03 - 0.10
More than one sign	79	43%	2	1.1%	38.6	66.6	<.0001	74	17.7 - 311
Total number	184		179						

*To prevent division by zero error, for each signs where no control manifest that sign, one dummy female control has been added positive for that sign, with mean values for age and date.

Training grade examiners reported fewer examinations where these signs were present than fully trained forensic paediatricians (cases: 68% versus 75%; controls 11% v 19%) but these differences were not significant (P = 0.4 and 0.2 respectively).

RAD and perianal venous congestion were seen commonly in cases but rarely or not at all in controls, resulting in high likelihood ratios. The estimated maximum horizontal diameter of dilatation was stated in 27 cases and was over 1 cm in 14 (52%) cases. Fissures and laxity were also seen more commonly in cases than controls. Anal tags were uncommon overall.

Of the less recognised signs, most were reported significantly more often in cases than controls (Table 3). Fold changes, were described fairly often, but the others were generally less common.

**Table 3:** Frequency of other anal signs not discussed by RCPCH (2008) in cases and controls

					**Unadjusted**			**Adjusted+**	
**Sign**	**Cases**		**Controls**		LR*	**OR**	**P**	**OR**	**95% CI**
Fold changes	34	18.5%	3	1.7%	10.9	13.3	<.0001	8.7	3.0 - 25
Twitching	17	9.2%	2	1.1%	8.4	9.1	<.0001	9.2	2 - 41
Swelling	12	6.5%	0	0	11.8	12.6	<0.001	15.4	1.9 - 120
Funnelling	8	4.3%	1	0.6%	7.2	8.1	0.037	6.4	0.75 - 53
Mucosal prolapse	8	4.3%	0	0	7.2	8.1	0.007	8.1	1.0 - 70
Abrasion	7	3.8%	0	0	6.9	7.1	0.015	10.6	1.2 - 90
Deficit	5	2.7%	0	0	NA		0.061		
Warts	1	0.5%	0	0	NA		1		
Soiling	5	2.7%	11	6.1%	NA		0.13		

*To prevent division by zero error, for each sign where no control manifest that sign, one dummy female control has been added positive for that sign, with mean values for age and date. This excludes variables with 5 or less positive in cases.

+Adjusted for date of exam, age and gender.

There were no significant effects of age or examiner grade on the prevalence of signs (data not shown) and simultaneous adjustment for age, gender and examination era made no meaningful difference to the results (Tables 2 and 3).

The prevalence of signs varied with interval to examination (Table 4). Erythema, swelling and fold changes occurred most commonly within 7 days of the alleged assault. RAD, laxity, venous congestion, fissure and twitching were seen up to 6 months.

**Table 4:** Anal findings in cases by time interval between last episode of abuse to examination

**Time since last assault**	**Unknown**	**<7 days**	**7 days to 6 months**	**>6 months**	P*
Reflex anal dilatation	17 (29%)	13 (22%)	9 (20%)	2 (9%)	0.21
Laxity	18 (31%)	15 (25%)	11 (25%)	5 (23%)	0.76
Reddening	21 (36%)	23 (39%)	11 (25%)	1 (4.3%)	0.002
Venous congestion	22 (38%)	23 (39%)	15 (34%)	6 (26%)	0.28
Fissure	5 (9%)	11 (19%)	9 (16%)	1 (4%)	0.21
Scar	6 (10.3%)	2 (3.4%)	2 (4.5%)	0	0.57
Any core sign	44 (76%)	48 (81%)	33 (75%)	11 (48%)	0.005
2 or more core signs	30 (52%)	30 (51%)	14 (32%)	5 (22%)	0.008
Fold changes	10 (17%)	15 (25%)	7 (16%)	2 (8.7%)	0.07
Twitching	2 (3.4%)	9 (15%)	4 (9.1%)	2 (8.7%)	0.32
Swelling	5 (8.6%)	6 (10%)	1 (2%)	0	0.04
Funnelling	2 (3.4%)	2 (3.4%)	3 (7%)	1 (4.3%)	0.69
Mucosal prolapse	4 (6.9%)	3 (5%)	0	1 (4.3%)	0.57
Abrasion	1 (2%)	6 (10%)	0	0	0.018
Total	54	58	55	17	

*χ^2^ trend excluding unknown.

History of constipation was recorded in 15 cases (7 boys, 8 girls), of whom 5 had RAD and 2 had fissures. There were 3 constipated controls (all girls) and each had one of venous congestion, a fissure and tag.

## Discussion

Martial wrote in 1^st^ century AD that “the favourite sexual use of children was not fellatio, but anal intercourse” [26]. Summit wrote “Manual, oral and anal containment of the penis are the “normal” activities of incestuous intercourse, as they are also for the more typically out of family sexual assault of boys” [27]. Anal signs were central in the Cleveland Inquiry [28] which recommended further study which in turn lead to publications by the Royal College of Physicians which provided guidance for clinicians [29,30]. Allegations of anal abuse appear to be relatively rare, as these disclosed cases represented only 5% of all CSA cases seen. This possibly explains why the recent RCPCH review noted a serious lack of evidence on anal signs in children [4]. The resulting uncertainty has limited doctor’s ability to provide clear opinions.

Identification of a group where CSA can be confidently diagnosed or excluded is always challenging. While we cannot be certain that all the children who alleged anal abuse were true cases, it is generally accepted that disclosure is strongly indicative of abuse. Ideally the non abused controls would be sampled from the general population, but in practice recruiting a truly representative group and excluding CSA can be problematic. Selection of children from the general population has proved quite difficult, but it also raises serious ethical considerations. In one of the few studies of this kind [15] only 10% of parents approached participated and some later admitted that concerns that their child had been abused motivated them to participate. In that study perianal venous congestion was more commonly seen (16%) than in another study where 1% of younger children showed this sign [14].

A different approach was used in a recent study [11]. Children evaluated for possible sexual abuse were divided into 2 groups, one with a low probability (917 children) and one with a high probability (198 children) of having been anally penetrated. Comparison was made between these groups in terms of the physical signs observed. However identifying comparison children with a low risk of having been anally penetrated in a group of children referred for sexual abuse evaluation is problematic as suggested by the presence of anal bruising in 10, anal fissure in 25 and anal laceration in 3. Consequently, the solution of choosing as controls children examined with concerns about other forms of abuse where the routine practice was to include anal examination seemed overall the best solution to us.

While physically abused and neglected children have a known increased risk of CSA [31], in this study the fact that wide ranging sensitive information was available minimised the likelihood of including unrecognised CSA. However, it is possible that an occasional sexually abused child could unintentionally have been included in the control group and if so this would mean that the prevalence of signs seen in the controls would be overestimated. Control children with anal photographs were more likely to be included in this study than those without, and this could also have had the effect of overestimating the proportion of controls with positive findings. If this were the case that would imply that the true difference between groups was in fact even greater.

There were small differences in examiner status between cases and controls, cases were drawn over a longer time period than controls and the age range of cases and controls was slightly different, but statistical adjustment for all these factors made no meaningful difference to the results.

An important remaining concern is the possibility of examiner bias. When examining a child who has alleged anal abuse, a physician might be more confident in reporting abnormal findings than in a child with no such history. However both groups were examined by the same staff who would be alert to the possibility of undisclosed anal abuse and with experience of eliciting the signs in question. This makes it possible that examiners in this centre were more likely to detect signs in general, but this would apply to both cases and controls.

Thus while the limitations of the samples must be recognised, this remains the first case/control study in which a large group of children all of whom disclosed anal abuse was examined using the same techniques and examiners as controls, using well defined terminology. The difference in frequency of some signs between cases and controls suggest that they are likely to relate to abuse. In particular RAD and perianal venous congestion were seen frequently in cases, but rarely or not at all in controls. RAD is dramatic, involves dilatation of both sphincters, requiring observation for up to 30 seconds as it does not always appear immediately. Previous studies found RAD in 10% to 34% disclosing anal abuse and 5% to 20% reporting any sexual abuse (Table 5) [4]. In children selected for non-abuse, RAD was noted in 5% examined in the knee chest, but less than 1% in the left lateral position [16]. Another study [15] found none with the sign. An earlier study which has influenced practice especially in North America [17] described anal dilatation in 49% children selected for non-abuse examined in the knee chest position, for up to 8 minutes. But this position is rarely used in the UK. Apart from that study our figures for cases (22%) and controls (0%) lie within the range of other studies for both abused and “non-abused” in the left lateral position.

**Table 5:** **Comparison with published studies reviewed by RCPCH**[4]

	**Cases**		**Controls**	
**Sign**	**This study**	**RCPCH review**	**This study**	**RCPCH review**
Reflex anal dilatation	22.3%	10 – 34% [5,6,8,10,11,13,32,33]	0%	<1 – 3.6% (left lateral) [11,14]
Laxity/reduced anal tone	26.6%	3 – 14% [7,12,34]	2.8%	No reports
Reddening/erythema	30.4%	1 – 12.6% [5-8,11,13,35]	8.3%	7 – 13.2% [11,13,14]
Perianal venous congestion	35.9%	8 – 36% [5,8,10,11,36]	0.6%	1% – 34.3% [11,13,14]
Fissure/laceration	14.1%	11 – 50% [5-7,10-12,32,35-38]	1.1%	1 – 3% [11,13,14]
Anal or perianal bruising	0%	0 – 10% [7,10,11,32]	0%	0 – 1.1% [11,14]
Any signs	74%	1 – 95% [5,6,32-35,39]	16%	No reports

Anal laxity (reduced anal tone) was seen more commonly in our cases than in earlier studies [7,12,36], but had never been previously considered in children selected for non-abuse (Table 5). Anal fissure and laceration are injuries in the perianal skin. There is a lack of agreed definitions to fully differentiate them. Our figures which combine fissures with lacerations gave prevalence for both cases and controls which were within the range described in other studies (Table 5). Perianal venous congestion was at the upper end of the range for cases in previous studies and the lower end for controls (Table 5). As with most previous studies, anal bruising was uncommon following abuse and rarely reported in “non-abuse”. Erythema was seen more commonly than in previous studies probably reflecting a higher proportion examined soon after an assault than in previous studies.

Anal dilatation and venous congestion were so rarely seen in controls, that it raises the possibility that they should be recognised as signs which should prompt further investigation, as long as they are interpreted in the broad context of a detailed medical, social and family assessment and the child’s behaviour and demeanour.

The highest frequency of signs was seen in those abused less than 7 days previously and in those where the timing of the abusive episode was not known. But none of the signs were seen only within 7 days of the alleged assault, suggesting that examination is worthwhile even some weeks after the alleged assault.

The majority of cases had at least one sign, though in many these were non-specific. This observation is consistent with previous studies reviewed by the RCPCH [4]. Of seven studies reporting any abnormal signs, two found these in 61-95% [5,35] and two in 46% and 57% [6,33], despite widely differing methodology and definitions. However a quarter had no signs, so the absence of physical signs could not be said to negate a child’s history or exclude the possibility of abuse.

## Conclusions

Anal physical findings in children are described following a disclosure of anal penetrative abuse. A majority of children who disclosed anal abuse had some signs, many of which were seen almost exclusively in cases and nearly half had multiple anal signs. Nearly half the cases had multiple anal signs compared to only 1% of controls. Reflex anal dilatation was seen in 22% cases but no controls.

This study strengthens understanding of physical signs following anal abuse and underlines the need for careful physical examination where this form of abuse is alleged by the child or suspected by those responsible for his protection. Anal findings thus have the potential to provide important corroboration of disclosed anal abuse.

## Competing interests

CJH is a retired NHS consultant who undertakes locum work that may involve child protection assessments and also provides expert medico legal opinions on Child Protection cases for which he receives a fee. CW is an honorary NHS consultant who advises on academic aspects of Child Protection and does not usually undertake paid medico legal work.

## Authors’ contributions

CJH was involved in: the conception, design, analysis and interpretation of data. Drafting the article and revising it critically for important intellectual content. Final approval of the version to be published. CMW was involved in: analysis and interpretation of data. Drafting the article and revising it critically for important intellectual content. Final approval of the version to be published. Both authors have given final approval for the publication of this manuscript.

## Authors’ information

CJH and CW are both involved with the Royal College of Paediatrics and Child Health Project on the Physical Signs of Child Sexual Abuse, CJH at one time was Chair of the Anal Working Group and CW is a member.

## Pre-publication history

The pre-publication history for this paper can be accessed here:

http://www.biomedcentral.com/1471-2431/14/128/prepub
